# Left Ventricular Thrombus With Silent Myocardial Infarction in a Patient With Factor V Leiden Mutation and High Factor VIII Levels: A Case Report

**DOI:** 10.7759/cureus.74944

**Published:** 2024-12-02

**Authors:** Abdul Samad Zahid, Mohammed Shariff, Robert Huggett

**Affiliations:** 1 Cardiology, Russells Hall Hospital, Dudley, GBR

**Keywords:** factor viii, factor v leiden, lv thrombus, silent mi, stroke

## Abstract

Silent myocardial infarction (SMI) is a type of myocardial infarction that occurs in the absence of, or with, minimal symptoms, often leading to a delay in medical treatment. There is a lack of data regarding the incidence and/or prevalence of a left ventricular (LV) thrombus in those who have had an SMI, due to the rarity of such cases. We describe a case of an SMI with LV thrombus in an otherwise healthy young man, whose first presentation was with stroke-type symptoms and who was also later found to have a Factor V Leiden (FVL) mutation and raised factor VIII levels. Further research is needed to investigate the relationship between these haematological conditions and the risk of arterial thrombosis including stroke, SMI, and LV thrombus.

## Introduction

Left ventricular (LV) thrombus is a common complication following an anterior ST-segment elevation myocardial infarction (STEMI) [1]. LV thrombus formation is also associated with reduced LV ejection fraction, wall motion abnormalities, and LV aneurysms [1]. Its association with silent myocardial infarction (SMI) is not well-documented. Factor V Leiden (FVL) is an autosomal dominant condition with incomplete penetrance that increases the risk of venous thromboembolism (VTE), although its association with arterial thromboembolism remains unclear [2]. Having FVL alone does not appear to increase the risk of developing arterial thrombosis, that is, heart attacks and strokes [3]. We report an unusual case of a young man, with no significant cardiovascular risk factors, found to have recurrent strokes, LV thrombus, and evidence of an SMI who was later discovered to have an FVL mutation and elevated factor VIII levels.

## Case presentation

A gentleman in his early 40s presented to the emergency department (ED) with right arm weakness and numbness followed by slurred speech, lasting approximately one hour. Two weeks prior, he had developed a severe headache associated with left-sided visual disturbances but did not seek medical attention. He denied chest pain, palpitations, syncope, or shortness of breath. He had no significant past medical history. His sister had a homozygous FVL mutation. He did not smoke cigarettes but had been smoking three to four roll-ups of cannabis mixed with tobacco per day for the past 15-20 years. He drank up to 30 units of alcohol per week. He worked in sales and led an active lifestyle. His diet consisted of mostly homecooked meals with occasional sugary snacks. His observations and examination (including cardiac and neurological) were normal. 

**Figure 1:**
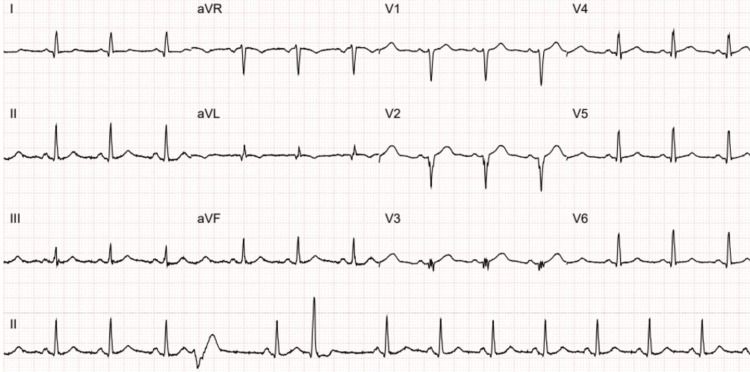
An electrocardiogram demonstrating sinus rhythm with multifocal premature ventricular complexes and pathological Q waves in leads V2 to V3

The computed tomography (CT) scan of his head showed no acute abnormalities. Initial blood tests were unremarkable other than the elevated triglyceride (15.1 mmol/L, normal <1.7 mmol/L) and low-density lipoprotein levels (4.6 mmol/L, normal <3.0 mmol/L). An electrocardiogram (ECG) showed sinus rhythm with multifocal premature ventricular complexes and pathological Q waves in leads V2 to V3 (Figure 1). He was seen by the stroke nurse in the ED and referred to the transient ischaemic attack (TIA) clinic. 

The next day, he was reviewed in the TIA clinic, and a magnetic resonance imaging (MRI) scan of the brain was performed on the same day. It was reported two weeks later and showed multifocal areas of recent infarction within the left middle cerebral artery territory as well as a chronic haemorrhagic infarct in the right medial occipital lobe. He was subsequently started on clopidogrel and atorvastatin by the stroke team.

**Figure 2:**
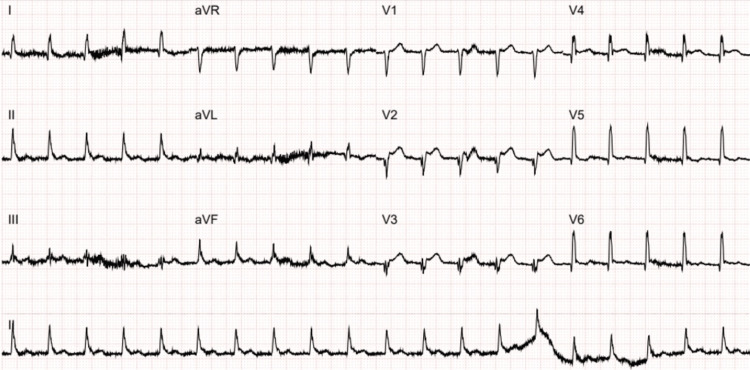
An electrocardiogram demonstrating <1 mm ST-segment elevation with pathological Q waves in leads V2 to V3

**Figure 3:**
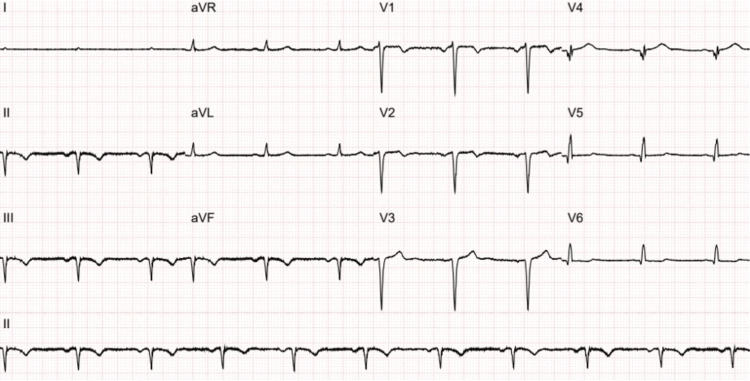
An electrocardiogram demonstrating new pathological Q waves and T wave inversion in leads II, III, and aVF

He re-presented to the ED approximately one month later with dizziness, blurred vision, and slurred speech. He denied any cardiac symptoms. Examination was normal. His ECG showed <1 mm ST-segment elevation with pathological Q waves in leads V2 to V3 (Figure 2). A repeat ECG 24 hours later showed new pathological Q waves and T wave inversion in leads II, III, and augmented vector foot (aVF) (Figure 3). There was no report of any chest pain. He was admitted onto the stroke ward, and a further MRI head did not show any new intracranial pathology. He was given a diagnosis of posterior TIA and was discharged with clopidogrel to take lifelong and a three-week course of aspirin. An outpatient ECG was also requested. 

**Figure 4:**
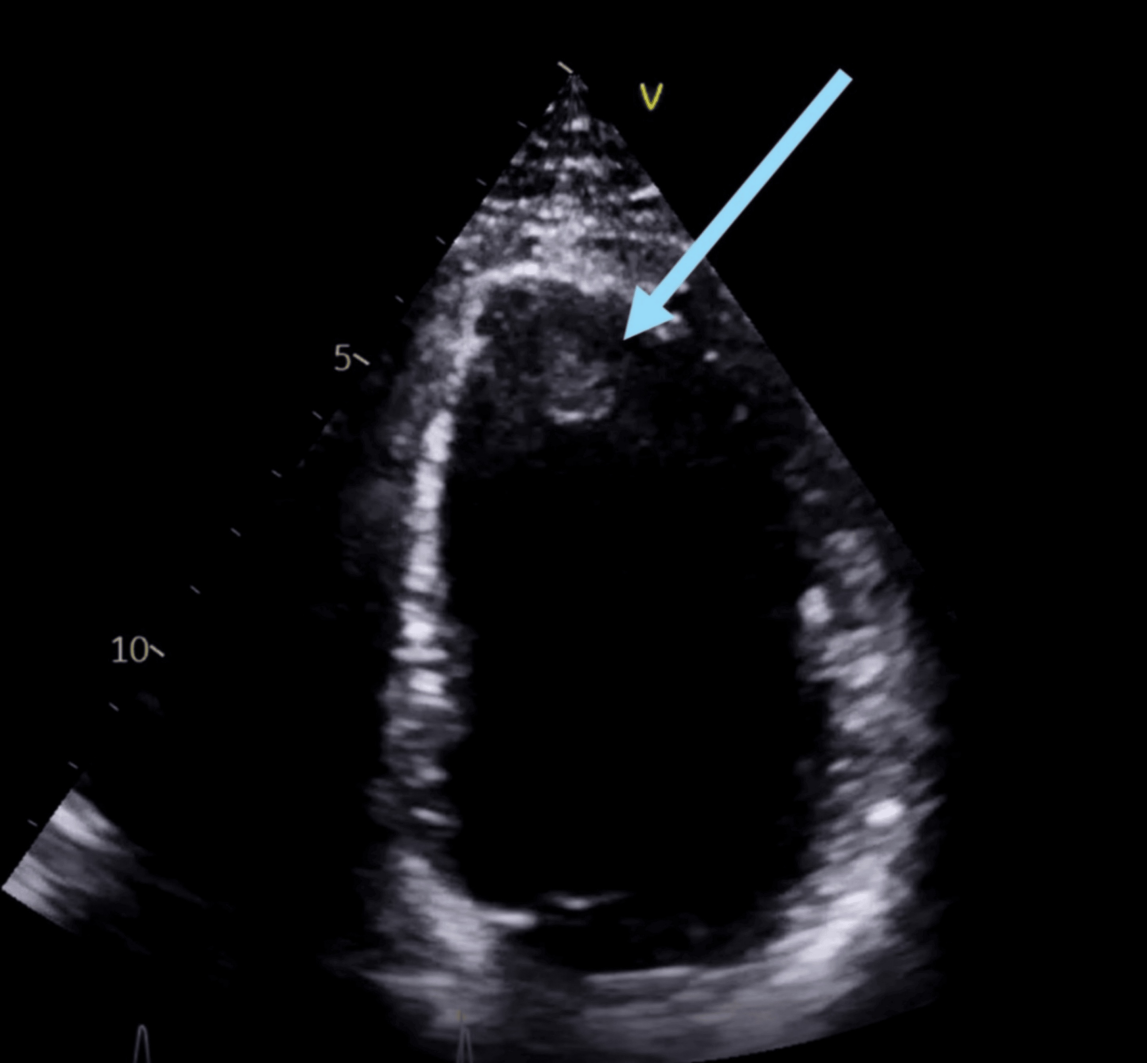
Transthoracic echocardiogram demonstrating a thrombus at the left ventricular apex (highlighted by blue arrow)

**Figure 5:**
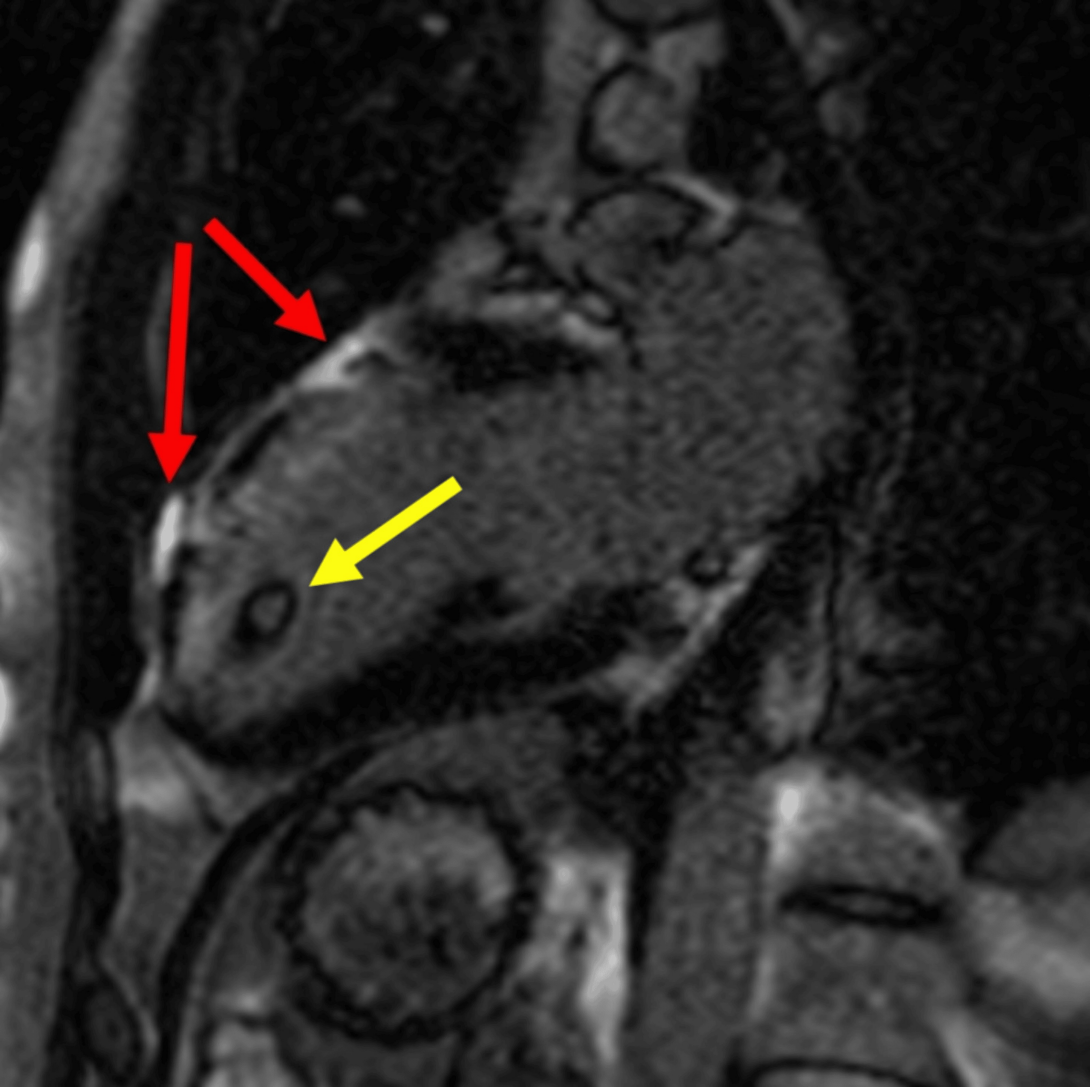
Cardiac magnetic resonance imaging scan (two-chamber view) demonstrating late gadolinium enhancement in the anterior segment of the heart, indicating left anterior descending artery territory infarction (highlighted by red arrows). A thrombus is also visible at the left ventricular apex (highlighted by yellow arrow)

Two months later, he attended for his outpatient ECG which showed a mildly dilated LV with impaired systolic function (ejection fraction: 45%-49%) in the presence of akinetic apical segments and an LV thrombus (Figure 4). He was subsequently admitted onto the cardiology ward. A young stroke screen done on his previous admission showed that the patient had a heterozygous FVL mutation and elevated levels of factor VIII and IX (both >150 IU/dL). Cardiac MRI confirmed moderately impaired LV systolic function due to a mature left anterior descending (LAD) artery territory myocardial infarction (MI) and a large mobile LV apical thrombus (16 x 8 mm) (Figure 5). CT coronary angiogram showed mild stenosis in the LAD artery due to diffuse non-calcified plaques. He was anticoagulated with rivaroxaban after consultation with the haematology team, and aspirin was also started after discussion with the stroke team due to the reduced risk of intracranial bleeding compared to clopidogrel. He was discharged with a plan for outpatient ECG in six months’ time. 

## Discussion

The incidence of LV thrombus after an anterior STEMI varies widely in reports from 4% to 39% [1]. LV thrombus is a serious complication of post-MI, associated with high rates of systemic embolism, morbidity, and mortality [4]. McCarthy et al. [4] screened 140,636 ECGs between January 2008 and May 2015, of which 0.09% were positive for an LV thrombus. It was found that the most common precipitating factor in those with an LV thrombus was heart failure (68.5%), followed by acute MI (25.9%); most of these cases were STEMI (71%) and/or involved the LAD artery (77.8%).

SMI prevalence is estimated to exist in 2% to 4% of the general population and is more frequent in men than in women [5]. There is little literature regarding the association between LV thrombus and SMI, likely due to the paucity of cases in clinical practice. Despite the rarity of SMI, an interesting case report on LV thrombus with SMI was reported by İnanç et al. [6], which described a young gentleman with 100% occlusion of his proximal LAD and a large, mobile apical thrombus. He underwent LV thrombectomy and coronary artery bypass surgery. A recent statement by the American Heart Association in 2021 [1] advises that there is currently insufficient data to recommend surgical excision of LV thrombi. They also state that direct oral anticoagulants such as rivaroxaban are a reasonable alternative to warfarin in patients with LV thrombus [1]. 

The patient described in the case report by İnanç et al. [6] was a heavy smoker, which may have contributed to the development of premature coronary artery disease. Although the patient in our case report did not smoke cigarettes, he did smoke cannabis, which has been associated with MI [7]; in particular, one case of SMI has been reported with the use of bonsai which is a synthetic derivative of cannabis [8]. Further research is needed to look at cannabis as an independent risk factor for MI [7]. The mechanism for cannabis-induced MI is likely multifactorial but may include increased autonomic activation, altered platelet aggregation, vasospasm, and toxic smoke constituents [7]. It should be noted that the patient in the case report by İnanç et al. [6] had breathing difficulties for one year, so one could debate if this was truly a silent MI. On the other hand, our patient had no cardiac symptoms, and his first symptoms reported were visual field disturbance, a presentation that has never been documented with SMI. 

FVL is an autosomal dominant condition with incomplete penetrance and is found in approximately 5% of the white population and is most common in people of Northern European descent and some Middle Eastern populations [2,3]. Heterozygous FVL mutations are associated with a sevenfold increase in the risk of VTE, whereas homozygous mutations (which are rare) increase the risk of VTE by 20-fold [2]. There is currently a lack of concrete evidence to suggest a significant relationship between FVL and arterial thromboembolic events, such as MI and ischaemic stroke [2], or between FVL and the development of LV thrombus in patients with an acute MI [9]. However, our patient had elevated levels of factor VIII which have been found to be associated with arterial thrombosis in both retrospective studies and case reports [10,11]. Interestingly, one report [11] describes a case similar to our own of a young man with obstructive disease in the LAD artery who also later developed ischaemic stroke. Testing for factor VIII levels is currently not recommended as part of a routine thrombophilia screen due to inconsistencies in sample analysis and interpretation [11]. 

## Conclusions

We have described a complex and rare case of a young patient presenting with stroke-like symptoms, who was later found to have an LV thrombus and evidence of a previous SMI, with a heterozygous FVL mutation and elevated factor VIII levels. We have not found any other reported cases of SMI with LV thrombus being associated with either FVL mutation, elevated factor VIII levels, or cannabis smoking. Although there are some documented cases of arterial thrombosis with high factor VIII levels, further studies are needed to investigate the relationship between the two and if factor VIII levels can be used to identify a subset of patients at high risk of thrombosis. Furthermore, the relationship between FVL and arterial thrombosis is even less well-established, and future studies may focus on looking at the relationship between these haematological disorders and, specifically, SMI and LV thrombus. Our patient had an SMI at an early age with dyslipidemia and cannabis as the only risk factors, which begs the question if FVL or high factor VIII levels had contributed to the underlying pathology.
